# Temperature Dysregulation Following Intrathecal Morphine for Cesarean Delivery Patients—A Scoping Review

**DOI:** 10.1111/aas.70350

**Published:** 2026-10-04

**Authors:** Christina Draegert, Pernille Pape, Heidi Kruse, Helene K. Nedergaard, Anne J. Wikkelsø

**Affiliations:** ^1^ Department of Anesthesiology and Intensive Care Zealand University Hospital Roskilde Denmark; ^2^ Department of Anesthesiology and Intensive Care Hvidovre University Hospital Hvidovre Denmark; ^3^ Department of Anesthesiology and Intensive Care University Hospital of Southern Denmark Kolding Denmark; ^4^ Department of Regional Health Research University of Southern Denmark Odense Denmark; ^5^ Department of Clinical Medicine University of Copenhagen Copenhagen Denmark

## Abstract

**Background:**

Intrathecal morphine is widely used for postoperative analgesia following cesarean delivery. However, intrathecal morphine's potential association with hypothermia remains uncertain, although hypothermia may adversely affect maternal recovery and comfort. This scoping review aims to map the available evidence regarding temperature dysregulation associated with intrathecal morphine in patients undergoing cesarean delivery.

**Methods:**

We conducted a scoping review according to the PRISMA‐ScR guideline. A systematic literature search was performed in Medline, Embase, The Cochrane Library, and Cinahl. Studies reporting temperature outcomes in patients receiving intrathecal morphine during cesarean delivery were eligible. The primary outcome was the incidence of hypothermia or hyperthermia. Secondary outcomes included changes in core temperature, dose–response relation, duration, severity of temperature dysregulation, and impact on recovery.

**Results:**

A total of 35 studies published between 1991 and 2025 were included. Four studies evaluated intrathecal morphine as intervention, whereas it was administered as standard care provided for all trial patients in the remaining studies. A total of 20 studies reported the incidence of hypothermia, corresponding to a pooled incidence of 21% among 773 patients. No studies reported an increase in temperature. A total of 12 case reports described severe hypothermia attributed to intrathecal morphine. However, our explorative analysis did not show an association between intrathecal morphine and hypothermia, and no dose response was identified. Clinical outcomes were infrequently reported, though several hypothermic patients were described as asymptomatic, suggesting that hypothermia may be clinically under‐recognized.

**Conclusion:**

Current evidence does not demonstrate a consistent association between intrathecal morphine and hypothermia following cesarean delivery. However, interpretation is limited by substantial heterogeneity across studies. This scoping review highlights an important evidence gap in the literature and supports the need for well‐designed studies to clarify whether intrathecal morphine contributes to clinically relevant hypothermia.

**Editorial Comment:**

This review presents an overview of the evidence concerning intrathecal morphine treatment and post‐caesarean temperature dysregulation. The evidence is incomplete, and this scoping review highlights areas where higher quality evidence is needed.

## 1 Introduction

Intrathecal morphine (ITM) is widely recommended for postoperative analgesia following cesarean delivery [1, 2, 3]. Intrathecal administration of morphine, a hydrophilic opioid, extends the duration of analgesia and may provide better postoperative pain relief compared to oral or systemic use [4]. However, the use of ITM is associated with side effects including pruritus, nausea, vomiting, urinary retention, and respiratory depression [5]. In addition, a possible morphine‐induced temperature dysregulation is reported as an adverse reaction but has received limited attention. Some case reports have described severe hypothermia attributed to intrathecal administration of morphine [6, 7], suggesting a contributing effect of morphine on body temperature dysregulation, especially hypothermia. The exact mechanism is not definitively known, but it is thought that the cephalic spread of morphine after intrathecal injection might alter the temperature threshold in the hypothalamus, thereby contributing to prolonged hypothermia [8]. Perioperative hypothermia is common under spinal anesthesia due to vasodilation and impaired thermoregulation [8, 9, 10]. Complications of hypothermia potentially include increased blood loss, compromised wound healing and infection, increased hospital stay, shivering, and patient discomfort [9]. Hypothermia during and after cesarean delivery affects maternal recovery and comfort, and maintenance of normothermia is recommended as part of the enhanced recovery protocols [2, 11].

### 1.1 Rationale

Temperature dysregulation caused by ITM remains uncertain. A comprehensive synthesis of existing clinical evidence is needed to guide enhanced recovery improvements and to identify possible knowledge gaps.

### 1.2 Objectives

This scoping review aims to provide an overview of the current evidence regarding the association between ITM and core temperature dysregulation (hypothermia or hyperthermia) in patients undergoing cesarean delivery with focus on incidence, onset, duration, severity, any dose–response phenomenon, and any possible impact on recovery.

## 2 Methods

### 2.1 Protocol and Registration

This scoping review was planned and reported in accordance with the Preferred Reporting Items for Systematic Review and Meta‐Analysis Protocol for Scoping Reviews (PRISMA‐ScR) guideline [12]. The protocol was prospectively registered at the Open Science Framework (https://osf.io/djfk6/overview).

### 2.2 Eligibility Criteria

Eligibility criteria were based on the PCC (population, concept, context) framework [13]. Population was defined as parturients, concept as ITM (any dose) and context was defined as cesarean delivery (scheduled or emergency) with measurement of core temperature. All clinical study designs were eligible, and we had no restrictions regarding publication year or language.

### 2.3 Information Sources and Search

The search strategy was developed in collaboration with a librarian experienced in database literature searches. We searched the databases of Medline, Embase, The Cochrane Library, and Cinahl. The initial search was carried out on November 13, 2024, and repeated on May 29, 2026 (Search strategy in Appendix S1). The reference lists of included studies were screened for additional relevant publications. References were imported to Covidence [14].

### 2.4 Selection of Sources of Evidence

Duplicates were removed, and two independent reviewers (CD and HK) screened titles and abstracts. Studies without clear information in the abstract regarding intrathecal medicine administered and temperature measurement were moved to full text screening. Two authors (CD and PP) subsequently evaluated relevant studies in full text. Studies with no description of temperature measurement were excluded. Any disagreements in screening were resolved through discussion between the screening authors and a third author.

### 2.5 Data Extraction and Qualitative Appraisal

Data extraction was performed independently by two authors (CD and PP) using pre‐piloted data extraction forms (Appendix S2). Each included study was evaluated for quality of evidence using the Mixed Methods Appraisal Tool (MMAT) [15] (Appendix S3). Case reports were automatically judged as very low quality. For studies not reporting essential data in the published article, we contacted the authors.

### 2.6 Data Items

Information extracted from each article included study characteristics, doses of intrathecal medicine including ITM administered, incidence of hypothermia or hyperthermia, lowest or highest mean (or median) core temperature measured respectively, time to onset and to recovery, and symptoms reported relevant to temperature dysregulation. If studies only reported temperature in a figure, we extracted the temperature from the figure to the best of our ability. The authors were approached to confirm that we extracted the precise temperature.

### 2.7 Outcomes

Primary outcome was temperature dysregulation defined as incidence of hypothermia (temperature < 36°C [16, 17] or as defined by authors) or hyperthermia (temperature > 38°C [18] or as defined by authors). The secondary outcomes were change in core temperature, dose‐relation, time to lowest/highest measured temperature, event severity, and impact on recovery.

### 2.8 Synthesis of Results

The extracted data from the included studies were synthesized in accordance with the defined outcomes. Quantitative data were presented in diagrammatic or tabular forms, while qualitative findings were summarized narratively. Included studies were categorized by whether ITM was administered as the intervention of interest or as part of standard care in trials investigating another intervention. Incidence of temperature dysregulation was assessed exploratively as a random‐effects meta‐analysis. Pooled incidence estimates with 95% confidence intervals were calculated using a random effects model in R version 4.6.1, package “meta”. Between‐study heterogeneity was assessed using the *I* ^2^ statistic and *τ* ^2^. Results were visualized as forest plots using *R*. For the studies comparing ITM with no ITM, we pooled extracted data on available outcome in meta‐analysis with a random effects model. Explorative analysis of plotted data on the possible correlation between dose of ITM and size of temperature decrease or time to lowest temperature following spinal anesthesia was performed as regression analysis with fitted linear regression of both Spearman and Pearsons in R using package “ggplot2”. If data were only available for separate groups in studies with ITM provided as standard, we used the placebo/standard group data for analysis.

## 3 Results

### 3.1 Selection of Studies

We identified 8368 relevant titles from the literature search, including citation searching, of which 2953 were removed as duplicates. We screened 5415 titles and abstracts, and 776 articles underwent full‐text review. Finally, 35 articles were included (Figure 1).

**FIGURE 1:**
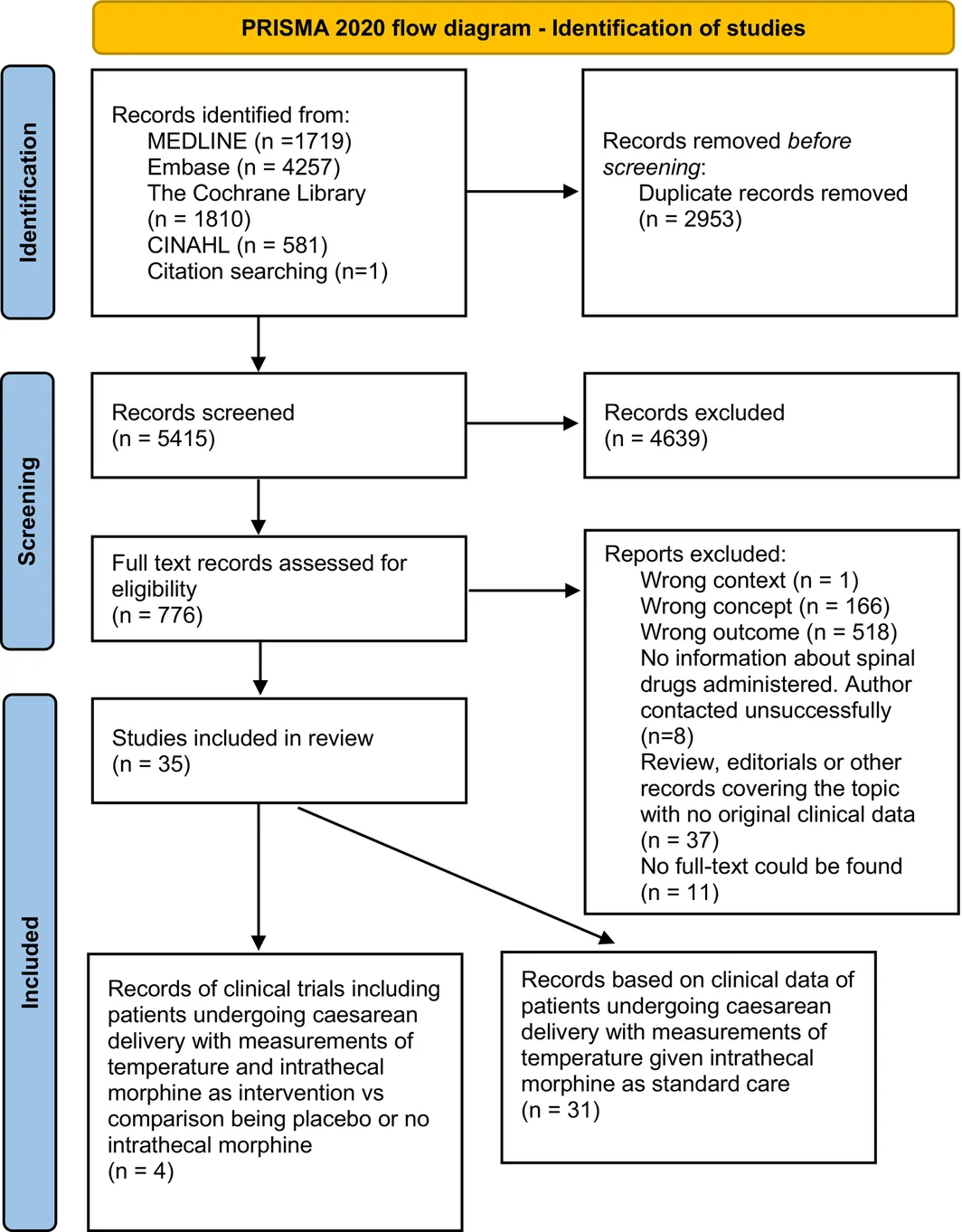
PRISMA flow diagram.

### 3.2 Characteristics of Included Studies

The 35 included studies were 13 randomized controlled trials (RCT) [19, 20, 21, 22, 23, 24, 25, 26, 27, 28, 29, 30, 31], one non‐randomized controlled trial [32], four prospective observational studies [33, 34, 35, 36], five retrospective observational studies [37, 38, 39, 40, 41], and 13 case reports or case series [33, 42, 43, 44, 45, 46, 47, 48, 49, 50, 51, 52, 53]. One study combined a case series with an observational design [33]. Only four studies (11%) investigated ITM as an intervention compared with a comparator group being placebo or no ITM [24, 25, 32, 40]. The remaining studies administered ITM as part of standard care with other interventions as focus. A total of 25 studies (71%) co‐administered a lipophilic opioid together with ITM and bupivacaine [20, 21, 22, 23, 27, 28, 29, 30, 31, 33, 34, 35, 36, 37, 38, 39, 40, 41, 42, 43, 45, 49, 50, 52, 53]. Seven studies (20%) had active warming as study focus [20, 21, 23, 26, 27, 38, 39]. In the remaining studies, excluding case reports, the study focus varied considerably. A total of 12 studies (34%) had temperature dysregulation, defined by author as incidence of hypothermia and/or decrease in core temperature, as primary outcome [20, 21, 23, 25, 27, 32, 33, 34, 37, 38, 39, 40]. In 11 studies (31%) core temperature was reported as a secondary outcome [19, 22, 24, 26, 28, 29, 30, 31, 35, 36, 41]. The method to measure core temperature varied among studies. A total of 13 studies (37%) measured core temperature by a tympanic device [19, 20, 24, 25, 27, 28, 30, 36, 40, 44, 45, 50, 52], 10 studies (29%) orally [21, 23, 31, 32, 33, 34, 35, 38, 43, 53], six studies (17%) rectally [42, 46, 48, 50, 51, 53], four studies (11%) by bladder [27, 35, 37, 38], three studies (9%) axillary [22, 42, 47], and three studies (9%) as temporal temperature [26, 35, 39]. Three studies (9%) did not report measurement method [29, 41, 49]. Overall, the included studies had a high degree of design heterogeneity. Study characteristics are summarized in Table 1.

**TABLE 1:** Study characteristics.

Study, year, country	Study design	Total patient No.	Role of ITM	Dose of ITM (μg)	Focus of study	Primary outcome
Hong, 2005, South Korea	RCT	119	Intervention	100 or 200	ITM/intrathecal Pethidin	Incidence of shivering
Hui, 2006, Taiwan	RCT	60	Intervention	150	ITM	Change in core temperature
Kavee, 1991, USA	Non‐randomized controlled trial	60	Intervention	500	ITM/epidural morphine	Change in core temperature
Munday…Yates 2018, Australia	Retrospective Observational	358	Intervention	0–200	ITM	Incidence of hypothermia
Hess, 2005, USA	Prospective, Observational	100	Standard care	250	ITM	Incidence of hypothermia
	Case series	14	Standard care	250	Cases of ITM‐induced hypothermia	
Abdel‐Ghaffar, 2019, Egypt	RCT	212	Standard care	200	Granisetron	Incidence of shivering
Bernardis, 2016, Brazil	RCT	40	Standard care	80	Active warming	Change in core temperature
Butwick, 2007, USA	RCT	30	Standard care	200	Active warming	Incidence of hypothermia
deFigueiredoLocks, 2012, Brazil	RCT	80	Standard care	80	Intrathecal Sufentanil	Incidence of shivering
Haim, 2024, Israel	RCT	66	Standard care	100	Active warming	Change in core temperature
Kholeif, 2024, Germany	RCT	60	Standard care	200	Active warming	Change in wound temperature
Munday…Gosden, 2018, Australia	RCT	50	Standard care	100	Active warming	Change in core temperature
Sadiq, 2025, Ireland	RCT	100	Standard care	N/R	Fasting protocols	Pre‐operative maternal comfort
Sween, 2021, USA	RCT	85	Standard care	250	Intravenous Dexmedetomi‐dine	Difference in patient‐rated shivering score
TanSookKuan, 2023, Malaysia	RCT	118	Standard care	100	Phenylephrine infusion	Incidence of shivering
Techanivate, 2005, Thailand	RCT	60	Standard care	200	Intrathecal Fentanyl	Incidence of shivering
Hilton, 2016, USA	Prospective, Observational	40	Standard care	200	Phenylephrine infusion	Change in core temperature
Ismaiel, 2025, USA	Prospective, Observational	30	Standard care	50–150	Temperature measurement modalities	Difference in temperature between modalities
Murphey, 2022, Ireland	Prospective Observational	33	Standard care	100	Infrared thermography	Change in cutaneous temperature
Burey, 2021, USA	Retrospective Observational	512	Standard care	200	Temperature monitoring with Foley Catheter	Change in core temperature
Hoefnagel, 2020, USA	Retrospective Observational	120	Standard care	150	Active warming	Change in core temperature
Marin, 2022, Germany	Retrospective Observational	111	Standard care	1 mcg/kg max 80	Active warming	Incidence of hypothermia
Riley, 2019, USA	Retrospective Observational	156	Standard care	200	Co‐loading with colloid versus crystalloid	Phenyl‐ephedrin administered
Bernstein, 2020, USA	Case report	1	Standard care	150	A case report of ITM‐induced hypothermia	
DeLeon, 2023, USA	Case report	1	Standard care	150	A case report of ITM‐induced hypothermia	
Fischer, 2006, France	Case report	1	Standard care	100	A case report of ITM‐induced hypothermia	
Harkouk, 2013, France	Case report	1	Standard care	50	A case report of ITM‐induced hypothermia	
Ioscovich, 2015, Israel	Case report	1	Standard care	150	A case report of ITM‐induced hypothermia	
Kanazawa, 2015, Japan	Case report	1	Standard care	1000	A case report of ITM‐induced hypothermia	
Mach, 2016, USA	Case report	1	Standard care	200	A case report of ITM‐induced hypothermia	
Munday, 2023, Australia	Case series	3	Standard care	100–200	A case report of ITM‐induced hypothermia	
Peillon, 2002, France	Case report	2	Standard care	100	A case report of ITM‐induced hypothermia	
Sayyid, 2003, Lebanon	Case report	1	Standard care	200	A case report of ITM‐induced hypothermia	
Wishaw, 1997, Australia	Case report	1	Standard care	250	A case report of ITM‐induced hypothermia	
Wolla, 2020, USA	Case report	1	Standard care	100	A case report of ITM‐induced hypothermia	

*Note:* Study characteristics of the included studies, with studies investigating ITM listed first, followed by the remaining studies grouped according to study design. ITM = intrathecal morphine, RCT = Randomized controlled trial.

### 3.3 Incidence of Temperature Dysregulation

A total of 22 (63%) of the included studies reported incidence of temperature dysregulation. In all studies, temperature dysregulation referred to hypothermia. No studies reported incidences of hyperthermia except Kavee et al. that specifically reported no events of hyperthermia from administration of 500 μg ITM [32]. A total of 12 of the 22 studies (55%) reporting on hypothermia were case reports or series [42, 43, 44, 45, 46, 47, 48, 49, 50, 51, 52, 53]. The remaining 10 studies included a total of 773 patients [21, 23, 27, 31, 33, 34, 35, 38, 39, 40]. The pooled incidence of hypothermia was 21% (CI 6%–56%) with considerable between‐study heterogeneity (I^2^ = 92,7%, τ^2^ = 4,65. *p* < 0,001) (Figure 2). This pooled incidence reflects the occurrence of hypothermia among patients receiving ITM and should not be interpreted as an ITM‐attributable risk, as the majority of contributing studies administered ITM as standard care to all patients and did not include a non‐ITM comparator group. Looking at outliers, two studies (Hilton et al. [34] and Techanivate et al. [31]) had zero events with ITM as standard care investigating change in core temperature or shivering from phenylephrine infusion or intrathecal fentanyl. The Hoefnagel et al. study found an 80% incidence of hypothermia in an observational design investigating active warming [38]. Sensitivity analysis omitting these three studies showed pooled incidence of 33% (CI 16%–56%), also with significant high heterogeneity.

**FIGURE 2:**
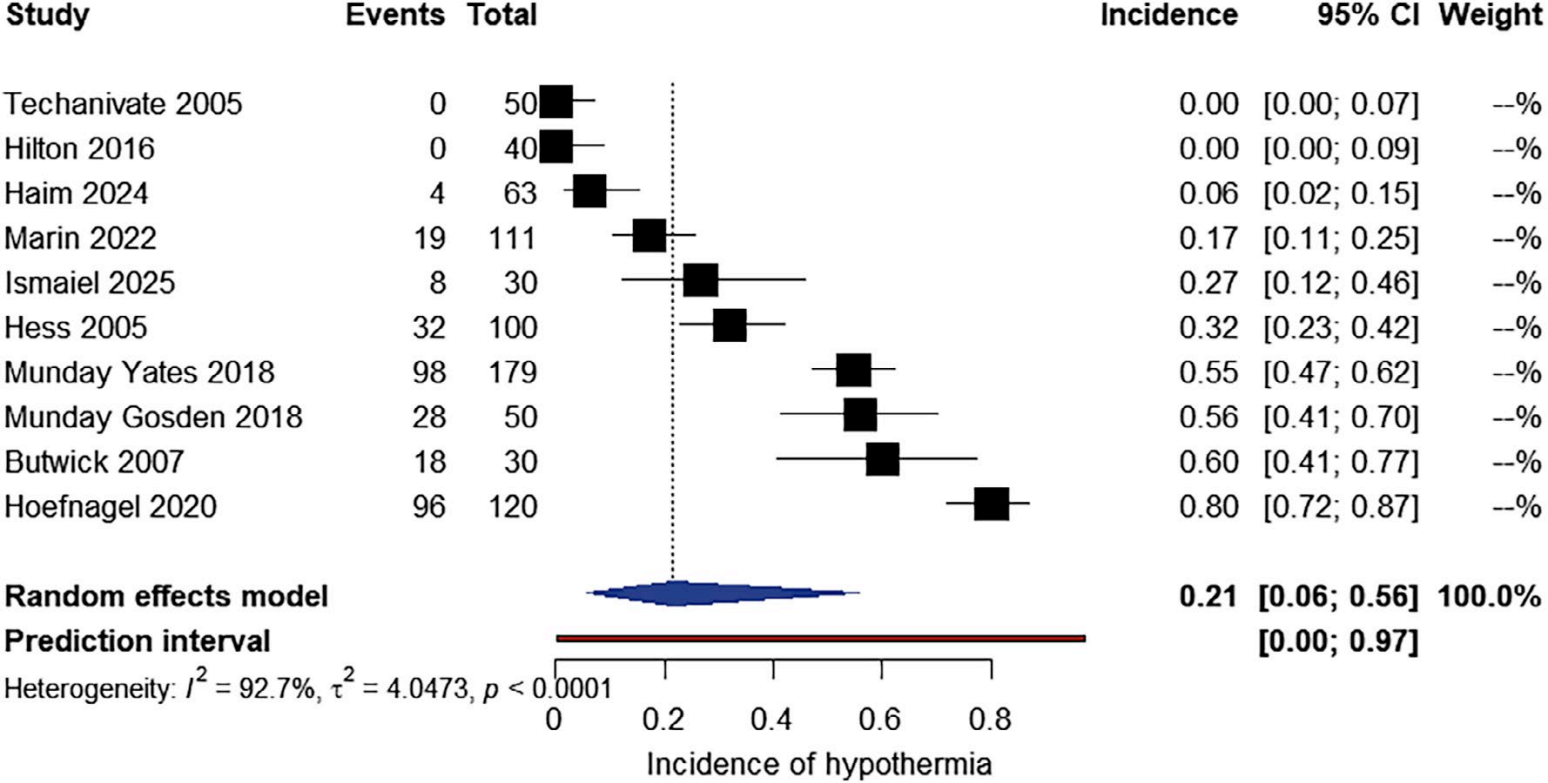
Incidence of hypothermia. Forest plot of the pooled incidence of hypothermia based on 10 studies reporting hypothermia incidence.

All included studies reported a post‐spinal anesthesia core temperature. Baseline measurements before spinal anesthesia were reported in 25 (71%) studies, all of which demonstrated a decrease in mean temperature following spinal anesthesia [19, 20, 21, 22, 23, 24, 25, 26, 27, 29, 30, 31, 32, 33, 34, 35, 36, 38, 39, 40, 41, 42, 48, 49, 53]. The extent of temperature decrease varied across studies, ranging from a difference of 0°C to −2.7°C.

Four studies investigated ITM in the dose range of 100–500 μg compared with a control group [24, 25, 32, 40]. However, only one of these, a retrospective observational study by Munday et al., reported incidence of hypothermia (55% and 58% for the ITM and control groups, respectively) with no significant difference between groups [40]. Similarly, the RCT by Hong et al. found no significant difference in temperature change between groups [24]. In contrast, Hui et al. and Kavee et al. both reported significantly greater reductions in core temperature among patients receiving ITM. The RCT by Hui et al. reported a maximum mean decrease in temperature of 1.11 (SD 0.61)°C compared with 0.76 (SD 0.39)°C in controls [25], whereas Kavee et al., a non‐randomized controlled study with the largest investigated ITM dose of 500 μg, reported a mean temperature decrease of 1.4 (SD 0.2)°C versus 0.8 (SD 0.13)°C, respectively [32]. Meta‐analysis of extracted data suggested a non‐significant trend toward greater temperature decline with ITM compared with no ITM (mean difference −0.26°C, CI −0.56, 0.03), but showed high heterogeneity (I^2^ = 92,6%) (Figure 3). The pooled estimate should be interpreted with caution given the substantial differences in study design, possible bias's and ITM dose across the included studies.

**FIGURE 3:**
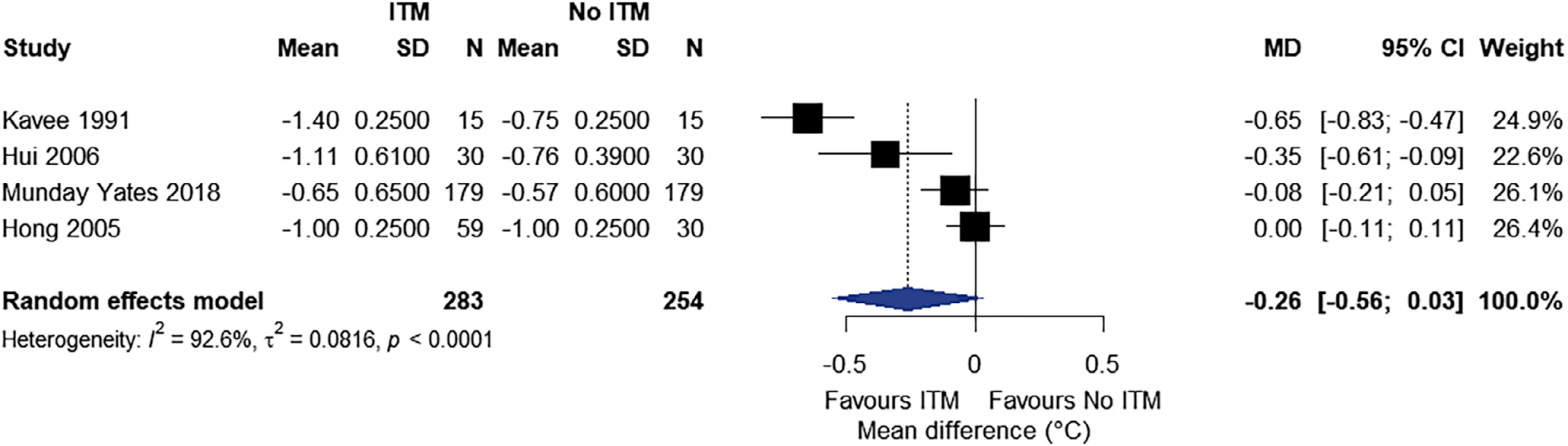
Temperature decline among studies investigating ITM. Meta‐analysis of extracted data on temperature decline following spinal anesthesia with ITM or no ITM. The pooled estimate includes data from studies with differing designs (randomized, non‐randomized, and retrospective observational) and varying ITM doses.

In the 12 case reports, the lowest temperature was 32.8°C–35.7°C with ITM doses ranging from 50–1000 μg.

### 3.4 Dose‐Relation and Time‐Course

There was a substantial variation in doses of ITM administered across studies, with most (71%) studies using doses in the range 100–200 μg [19, 21, 23, 24, 25, 26, 27, 30, 31, 34, 35, 36, 37, 38, 40, 41, 42, 43, 44, 46, 48, 49, 50, 51, 53]. In one case report, 1000 μg ITM was administered by accident [47]. The patient's core temperature decreased to 34°C, which was not lower than the minimum temperatures reported in other case reports despite substantially lower ITM doses. Exploratory pooled regression analysis found no association between ITM dose and maximum decrease in temperature following spinal anesthesia (*p* = 0.852; Pearson's *r* = 0.037; R^2^ = 0.001).

Among studies reporting hypothermia incidence, hypothermic events were identified either during the perioperative period or in the post‐anesthesia care unit. A precise onset time of hypothermia was not reported. The time interval from spinal anesthesia to the lowest recorded temperature ranged from 12–262 min across studies. Explorative analysis of data from studies reporting on time to lowest temperature showed no correlation between ITM dose and time to lowest temperature (*p* = 0.210; Pearson's *r* = 0.24; R^2^ = 0.056).

### 3.5 Severity of the Event and Impact on Recovery

The most frequently reported symptom associated with hypothermia was shivering. Additional symptoms were nausea, vomiting, pruritus, diaphoresis, and ‘feeling hot’. Some cases also reported dizziness and somnolence. Notably, a substantial proportion of patients were asymptomatic. One study reported an overall incidence of hypothermia of 32%, whereas the incidence of symptomatic hypothermia was only 6% [33]. However, in most of the studies included, subjective and objective symptoms were not reported. One study described a single case with wound infection among 50 patients [27]. None of the remaining studies identified a significant clinical impact of hypothermia in terms of prolonged hospital stay or postoperative infections. Overall, the impact on recovery was generally not well described.

### 3.6 Quality Appraisal

Using the MMAT, five studies met all five methodological quality criteria [19, 23, 25, 30, 36], while 70% of studies met at least four of the five criteria (≥ 80%) [19, 21, 23, 24, 25, 26, 27, 29, 30, 31, 33, 34, 35, 36, 39, 41]. Several studies were marked “can't tell” for one or more criteria, primarily reflecting incomplete reporting rather than clear methodological limitations. The quality appraisal scores are presented in Table 2. For detailed scoring see Appendix S3. Overall, the body of evidence should be interpreted with caution. Most included studies (63%) were non‐randomized, including 12 case reports, limiting the overall strength of the available evidence.

**TABLE 2:** Quality appraisal.

Study	Quality criteria met
Quantitative randomized controlled trials	
Abdel‐Ghaffar, 2019	100%
Bernardis, 2016	60%
Butwick, 2007	80%
deFigueiredoLocks, 2012	60%
Haim, 2024	100%
Hong, 2005	80%
Hui, 2006	100%
Kholeif, 2024	80%
Munday…Gosden, 2018	80%
Sadiq, 2025	60%
Sween, 2021	80%
TanSookKuan, 2023	100%
Techanivate, 2005	80%
Quantitative non‐randomized	
Hoefnagel, 2020	60%
Ismaiel, 2025	80%
Kavee, 1991	20%
Marin, 2022	80%
Munday…Yates 2018	60%
Riley, 2019	80%
Quantitative descriptive	
Burey, 2021	40%
Hess, 2005	80%
Hilton, 2016	80%
Munday, 2023	20%
Murphey, 2022	100%

*Note:* Quality appraisal of each study using the Mixed Methods Appraisal Tool. A total of 0 criteria met = 0%, 1 criteria met = 20%, 2 criteria met = 40%, 3 criteria met = 60%, 4 criteria met 80%, all 5 criteria met = 100%.

## 4 Discussion

In this scoping review of temperature dysregulation related to ITM in patients undergoing cesarean delivery, we identified 35 articles published between 1991 and 2025. Only four studies investigated ITM as an intervention, whereas the remaining studies administered ITM as standard care provided for all trial patients. Incidence of hypothermia was reported in 20 studies, resulting in a pooled incidence of 21% among 773 patients. Importantly, this estimate should not be interpreted as an ITM‐attributable risk, as most contributing studies did not include a non‐ITM comparator group. No correlation was found between dose of ITM and either change in core temperature or the time to the lowest recorded temperature.

Core body temperature is tightly regulated by hypothalamic thermoregulatory mechanisms, with decreased temperature triggering vasoconstriction and shivering. Spinal anesthesia impairs these cold‐defense responses in blocked areas, making hypothermia common in un‐warmed patients and typically lowering core temperature by 1°C–2°C [54]. Opioids are thought to lower the threshold for cold‐defense responses, thereby exacerbating spinal‐induced hypothermia [55]. Morphine is particularly relevant because of its hydrophilic properties, which cause slow spinal cord penetration and prolonged duration in the cerebrospinal fluid [8]. This results in a slow onset and prolonged duration of action up to 24 h. These pharmacokinetic characteristics provide a plausible rationale for delayed and prolonged thermoregulatory effects following ITM administration [56, 57]. Lipophilic opioids are also thought to modify thermoregulation, particularly by suppressing shivering, and may therefore represent a potential confounding factor when assessing ITM‐associated hypothermia. However, the hypothermic effect of lipophilic opioids is not well described [58, 59].

Despite this theoretical mechanism, our findings do not suggest a higher overall incidence of hypothermia with ITM than the 30%–60% commonly reported after cesarean delivery under spinal anesthesia [9, 60, 61, 62]. Among the four included studies comparing ITM with no ITM, results were conflicting, and an explorative meta‐analysis found no significant association between ITM and temperature decrease. Kavee et al. investigated the large dose of 500 μg and demonstrated that core temperature reached its lowest point approximately 2 h after spinal anesthesia and remained lower than in controls for up to 24 h [32], supporting the hypothesis that any thermoregulatory effect of ITM may have a delayed onset and prolonged duration. By comparison, the remaining comparative studies monitored temperature for only 60–90 min and may therefore have missed delayed temperature changes. More broadly, only 18 included studies (51%) monitored temperature beyond two hours [21, 23, 31, 32, 33, 39, 42, 43, 44, 45, 46, 47, 48, 49, 50, 51, 52, 53], decreasing to 26% after excluding case reports. Consequently, incidence of hypothermia or decrease in temperature associated with ITM may be underestimated in the current literature.

Low environmental temperature may accentuate the hypothermic effect in some animals [63], and data obtained from rats suggests that stress from restraint may also change the hypothermic response of opioids [64]. Whether these factors contribute to the heterogeneity and pooled results in human parturients remains unknown.

Another important source of heterogeneity was the method used to measure temperature. While tympanic, oral, bladder, and axillary measurements can provide acceptable estimates of core temperature when performed appropriately, skin‐based measurements are generally less reliable [17]. Ismail et al. demonstrated significant differences in measured temperatures and hypothermia incidence depending on the monitoring site, with poor agreement between temporal and sublingual measurements [35]. Although 80% of the studies included in this review used methods generally considered appropriate for estimating core temperature [19, 20, 21, 22, 23, 24, 25, 27, 28, 30, 31, 32, 33, 34, 35, 36, 37, 38, 39, 40, 42, 43, 44, 45, 47, 50, 52, 53], the absence of a standardized monitoring approach introduces measurement variability and may have contributed to inconsistent findings across studies.

The clinical importance of hypothermia depends not only on its incidence but also on its impact on patient outcomes. Although perioperative hypothermia has been associated with adverse outcomes such as increased blood loss, impaired wound healing, surgical site infection, shivering, and patient discomfort [9], these outcomes were rarely reported in the included studies. However, it remains unclear whether this reflects incomplete reporting or a genuinely low symptom burden. One study found that 81% of hypothermic patients remained asymptomatic [33], consistent with the hypothesis that neuraxial anesthesia may reduce the perception of cold and associated discomfort [54]. Several case reports described paradoxical clinical manifestations of hypothermia like feeling hot and being diaphoretic, suggesting that hypothermia following ITM may present atypically. No severe hypothermia‐related complications were identified, although this should be interpreted cautiously as follow‐up generally was limited to the post‐anesthesia care unit.

## 5 Limitations

This scoping review has several limitations. Considerable heterogeneity existed across the included studies regarding study design and focus, ITM dose, temperature monitoring method, perioperative warming practices, and outcome reporting, limiting comparability. Only four studies specifically investigated ITM, and hypothermia was rarely a primary outcome, making it difficult to determine whether ITM independently contributes to hypothermia. Furthermore, missing and incompletely reported data represented an important limitation, and analyses are based on pooled results not on individual data. Finally, 63% of the included studies were non‐randomized designs, resulting in an overall lower level of evidence and increased risk of bias. As a scoping review, the objective was to map the available literature rather than formally assess study quality or estimate treatment effects. Therefore, the findings should be interpreted as an overview of the existing evidence.

## 6 Conclusion

Hypothermia is common among patients undergoing cesarean delivery with ITM, with a pooled incidence of approximately 21%. However, the available evidence does not demonstrate a consistent association between ITM and hypothermia or any dose response. Interpretation is limited by substantial heterogeneity across studies and limited duration of temperature monitoring, which may have underestimated delayed temperature changes associated with ITM. Furthermore, the clinical significance of hypothermia remains uncertain, as patient symptoms and recovery outcomes were infrequently reported. Overall, this scoping review demonstrates an important gap in evidence, and well‐designed studies are needed to clarify whether ITM contributes to clinically relevant hypothermia following cesarean delivery.

## Author Contributions

**Christina Draegert:** study conception, article screening, data extraction, writing of the draft, draft revision. **Helene K. Nedergaard:** study conception. **Anne J. Wikkelsø:** study conception. **Pernille Pape:** article screening, data extraction. All four authors have assisted in revising and subsequently approving the final manuscript.

## Funding

The authors have nothing to report.

## Conflicts of Interest

The authors declare no conflicts of interest.

## Supporting information


**Appendix S1:** Search strategy in Medline, Embase, The Cochrane Library and Cinahl.

**Appendix S2:** Covidence Data Extraction Template.

**Appendix S3:** Quality appraisal using Mixed Method Appraisal Tool (MMAT).

## Data Availability

The data that support the findings of this study are available from the corresponding author upon reasonable request.
