# Correction to “Temperature Dysregulation Following Intrathecal Morphine for Cesarean Delivery Patients—A Scoping Review”

**DOI:** 10.1111/aas.70366

**Published:** 2026-10-26

C. Draegert , P. Pape , H. Kruse , H. K. Nedergaard , and A. J. Wikkelsø , “Temperature Dysregulation Following Intrathecal Morphine for Cesarean Delivery Patients—A Scoping Review,” Acta Anaesthesiologica Scandinavica 70, no. 10 (2026): e70350, 10.1111/aas.70350.PMC1363468442830199

In the Author Contributions section, one of the author's contributions was inadvertently omitted. The Author Contributions section should have included all five authors and should have read as follows: “**Christina Draegert:** study conception, article screening, data extraction, writing of the draft, draft revision. **Helene K. Nedergaard:** study conception. **Anne J. Wikkelsø:** study conception. **Pernille Pape:** article screening, data extraction. **Heidi Kruse:** article screening. All five authors have assisted in revising and subsequently approving the final manuscript.”

We apologize for this error.

