# Supplementary material for: Temperature Dysregulation Following Intrathecal Morphine for Cesarean Delivery Patients—A Scoping Review
Source: Acta Anaesthesiol Scand. 2026 Oct 4;70(10):e70350. doi: 10.1111/aas.70350 (PMC13634684; doi:10.1111/aas.70350)
Supplement: Supplementary file 1 [file AAS-70-0-s001.docx]

**Appendix S1**
Search strategy in Medline, Embase, The Cochrane Library and Cinahl

**Medline**
Database: Ovid MEDLINE(R) ALL <1946 to November 12, 2024>

| **#** | **Query** | **Results from 13 Nov 2024** |
| --- | --- | --- |
| 1 | Analgesics, Opioid/ | 64,942 |
| 2 | Morphine/ | 41,056 |
| 3 | 1 or 2 | 94,121 |
| 4 | (morphine or morphia or duramorph or opioid* or opiate*).ab,kf,ti. | 177,183 |
| 5 | 3 or 4 | 195,465 |
| 6 | Injections, Spinal/ | 13,416 |
| 7 | Anesthesia, Spinal/ | 13,442 |
| 8 | Anesthesia, Obstetrical/ | 13,735 |
| 9 | Subarachnoid Space/ | 4,634 |
| 10 | 6 or 7 or 8 or 9 | 40,828 |
| 11 | (spinal* or intraspinal* or "intra spinal*" or intrathecal* or "intra thecal*" or neuraxial* or neuroaxial* or subarachnoid* or "obstetric* anesthesia" or "obstetric* anaesthesia" or "delivery anaesthesia" or "delivery anesthesia").ab,kf,ti. | 398,450 |
| 12 | 10 or 11 | 413,097 |
| 13 | Cesarean Section/ | 54,819 |
| 14 | (caesarean or cesarean or "c section*" or "abdominal deliver*" or postcaesarean or postcesarean or caesarian or cesarian).ab,kf,ti. | 81,115 |
| 15 | 13 or 14 | 93,140 |
| 16 | Body Temperature/ | 50,013 |
| 17 | Hypothermia/ | 15,369 |
| 18 | Hyperthermia/ | 423 |
| 19 | Fever/ | 44,315 |
| 20 | Rewarming/ | 1,676 |
| 21 | 16 or 17 or 18 or 19 or 20 | 103,958 |
| 22 | (temperature* or rewarm* or "warming up" or "warming techniques" or hypotherm* or hypertherm* or fever or normotherm*).ab,kf,ti. | 1,149,772 |
| 23 | 21 or 22 | 1,179,008 |
| 24 | 5 and 12 and 15 | 1,360 |
| 25 | 12 and 15 and 23 | 256 |
| 26 | 24 or 25 | 1,571 |

**Embase**

Database: Embase <1974 to 2024 November 12>

| **#** | **Query** | **Results from 13 Nov 2024** |
| --- | --- | --- |
| 1 | morphine/ | 120,594 |
| 2 | opiate/ | 121,545 |
| 3 | narcotic analgesic agent/ | 29,887 |
| 4 | 1 or 2 or 3 | 238,171 |
| 5 | (morphine or morphia or duramorph or opioid* or opiate*).ab,kf,ti. | 243,382 |
| 6 | 4 or 5 | 328,843 |
| 7 | intraspinal drug administration/ | 2,480 |
| 8 | spinal anesthesia/ | 29,406 |
| 9 | subarachnoid space/ | 8,292 |
| 10 | obstetric anesthesia/ | 9,181 |
| 11 | 7 or 8 or 9 or 10 | 46,389 |
| 12 | (spinal* or intraspinal* or "intra spinal*" or intrathecal* or "intra thecal*" or neuraxial* or neuroaxial* or subarachnoid* or "obstetric* anesthesia" or "obstetric* anaesthesia" or "delivery anaesthesia" or "delivery anesthesia").ab,kf,ti. | 515,584 |
| 13 | 11 or 12 | 530,331 |
| 14 | cesarean section/ | 135,153 |
| 15 | (caesarean or cesarean or "c section*" or "abdominal deliver*" or postcaesarean or postcesarean or caesarian or cesarian).ab,kf,ti. | 119,588 |
| 16 | 14 or 15 | 159,952 |
| 17 | body temperature/ | 65,032 |
| 18 | hypothermia/ | 40,620 |
| 19 | inadvertent perioperative hypothermia/ | 90 |
| 20 | hyperthermia/ | 26,354 |
| 21 | fever/ | 356,174 |
| 22 | warming/ | 16,032 |
| 23 | 17 or 18 or 19 or 20 or 21 or 22 | 479,213 |
| 24 | (temperature* or rewarm* or "warming up" or "warming techniques" or hypotherm* or hypertherm* or normotherm*).ab,kf,ti. | 958,219 |
| 25 | 23 or 24 | 1,335,841 |
| 26 | 6 and 13 and 16 | 2,818 |
| 27 | 13 and 16 and 25 | 621 |
| 28 | 26 or 27 | 3,322 |

**The Cochrane Library**

Date Run: 13/11/2024 12:56:10

| **ID** | **Search** | **Hits** |
| --- | --- | --- |
| #1 | MeSH descriptor: [Analgesics, Opioid] this term only | 10939 |
| #2 | MeSH descriptor: [Morphine] explode all trees | 6082 |
| #3 | #1 or #2 | 14037 |
| #4 | (morphine or morphia or duramorph or opioid* or opiate*):ti,ab,kw (Word variations have been searched) | 46497 |
| #5 | #3 or #4 | 46497 |
| #6 | MeSH descriptor: [Injections, Spinal] this term only | 1220 |
| #7 | MeSH descriptor: [Anesthesia, Spinal] this term only | 2953 |
| #8 | MeSH descriptor: [Anesthesia, Obstetrical] this term only | 1527 |
| #9 | MeSH descriptor: [Subarachnoid Space] this term only | 185 |
| #10 | #6 or #7 or #8 or #9 | 4741 |
| #11 | (spinal* or intraspinal* or intra NEXT spinal* or intrathecal* or intra NEXT thecal* or neuraxial* or neuroaxial* or subarachnoid* or obstetric* NEXT anesthesia or obstetric* NEXT anaesthesia or delivery NEXT anaesthesia or delivery NEXT anesthesia):ti,ab,kw (Word variations have been searched) | 45191 |
| #12 | #10 or #11 | 45910 |
| #13 | MeSH descriptor: [Cesarean Section] this term only | 4592 |
| #14 | (caesarean or cesarean or c NEXT section* or abdominal NEXT deliver* or postcaesarean or postcesarean or caesarian or cesarian):ti,ab,kw (Word variations have been searched) | 18813 |
| #15 | #13 or #14 | 18813 |
| #16 | MeSH descriptor: [Body Temperature] this term only | 2776 |
| #17 | MeSH descriptor: [Hypothermia] this term only | 971 |
| #18 | MeSH descriptor: [Hyperthermia] this term only | 128 |
| #19 | MeSH descriptor: [Fever] this term only | 2536 |
| #20 | MeSH descriptor: [Rewarming] this term only | 214 |
| #21 | #16 or #17 or #18 or #19 or #20 | 5893 |
| #22 | (temperature* or rewarm* or warming NEXT up or warming NEXT techniques or hypotherm* or hypertherm* or normotherm*):ti,ab,kw (Word variations have been searched) | 39105 |
| #23 | #21 or #22 | 40910 |
| #24 | #5 AND #12 AND #15 | 1381 |
| #25 | #12 AND #15 AND #23 | 231 |
| #26 | #24 OR #25 | 1575 |

**Cinahl**

Database: CINAHL with Full Text, Wed, November 13, 2024

Wed, November 13, 2024 03:05:40 pm

| **#** | **Query** | **Results** |
| --- | --- | --- |
| S52 | S50 OR S51 | 530 |
| S51 | S23 AND S34 AND S49 | 73 |
| S50 | S5 AND S23 AND S34 | 467 |
| S49 | S40 OR S48 | 63,580 |
| S48 | S41 OR S42 OR S43 OR S44 OR S45 OR S46 OR S47 | 52,402 |
| S47 | "normotherm*" | 1,528 |
| S46 | "hypertherm*" | 4,861 |
| S45 | "hypotherm*" | 12,081 |
| S44 | "warming techniques" | 1,459 |
| S43 | "warming up" | 814 |
| S42 | "rewarm*" | 1,059 |
| S41 | "temperature*" | 39,599 |
| S40 | S35 OR S36 OR S37 OR S38 OR S39 | 21,992 |
| S39 | (MH "Warming Techniques") | 1,446 |
| S38 | (MH "Fever") | 13,198 |
| S37 | (MH "Hypothermia") | 4,258 |
| S36 | (MH "Hyperthermia") | 179 |
| S35 | (MH "Body Temperature") | 4,935 |
| S34 | S24 OR S33 | 35,143 |
| S33 | S25 OR S26 OR S27 OR S28 OR S29 OR S30 OR S31 OR S32 | 35,143 |
| S32 | "cesarian" | 11,510 |
| S31 | "caesarian" | 260 |
| S30 | "postcesarean" | 184 |
| S29 | "postcaesarean" | 23 |
| S28 | "abdominal deliver*" | 63 |
| S27 | "c section*" | 11,966 |
| S26 | "cesarean" | 31,136 |
| S25 | "caesarean" | 9,368 |
| S24 | (MH "Cesarean Section") | 21,819 |
| S23 | S9 OR S22 | 118,047 |
| S22 | S10 OR S11 OR S12 OR S13 OR S14 OR S15 OR S16 OR S17 OR S18 OR S19 OR S20 OR S21 | 117,520 |
| S21 | "delivery anesthesia" | 17 |
| S20 | "delivery anaesthesia" | 2 |
| S19 | "obstetric* anaesthesia" | 278 |
| S18 | "obstetric* anesthesia" | 2,741 |
| S17 | "subarachnoid*" | 8,965 |
| S16 | "neuroaxial*" | 64 |
| S15 | "neuraxial*" | 1,458 |
| S14 | "intra thecal*" | 8 |
| S13 | "intrathecal*" | 5,151 |
| S12 | "intra spinal*" | 19 |
| S11 | "intraspinal*" | 3,788 |
| S10 | "spinal*" | 103,523 |
| S4 | "morphine” or “morphia” or “duramorph” or “opioid*” or “opiate*" | 65,636 |
| S3 | S1 OR S2 | 30,385 |
| S2 | (MH "Analgesics, Opioid") | 25,278 |
| S1 | (MH "Morphine") | 7,994 |
