# Supplementary material for: Temperature Dysregulation Following Intrathecal Morphine for Cesarean Delivery Patients—A Scoping Review
Source: Acta Anaesthesiol Scand. 2026 Oct 4;70(10):e70350. doi: 10.1111/aas.70350 (PMC13634684; doi:10.1111/aas.70350)
Supplement: Supplementary file 2 [file AAS-70-0-s002.docx]

**Appendix S2**

Covidence Data Extraction Template

General information

- Lead author contact details
- Title
- Year of publication
- Country of origin

Methods

- Aim of study
- Study design
- Does the study have intrathecal morphine as the intervention? (yes/no)
  - If yes, does the study have a comparison group?
  - If no, in what context is intrathecal morphine given?
  - If no, what is the intervention? And what is the comparison group, if any?
- Is temperature disorder the primary outcome? (yes/no)
  - If no, what is the primary outcome?
- Inclusion and exclusion criteria

Population

- Total number of participants
  - No. of participants in intervention group
  - No. of participants in comparison group, if any
- Baseline temperature

Concept

- Dose of intrathecal morphine administered
- Other local anesthetics or opioids administered
- Prophylactic measures to avoid temperature dysregulation

Outcome

- Is hypothermia and/or hypothermia defined by author?
- No. of patients reported with hypothermia or hyperthermia
- Lowest (if hypothermia) or highest (if hyperthermia) temperature measured after intervention
- Time to measured temperature after intervention
- Time to normothermia
- Method to measure temperature
- Duration of surgery
- Estimated blood loss
- Symptoms relevant to temperature dysregulation reported
- Reported impact of recovery
- Treatment given
