# Supplementary material for: Temperature Dysregulation Following Intrathecal Morphine for Cesarean Delivery Patients—A Scoping Review
Source: Acta Anaesthesiol Scand. 2026 Oct 4;70(10):e70350. doi: 10.1111/aas.70350 (PMC13634684; doi:10.1111/aas.70350)
Supplement: Supplementary file 3 [file AAS-70-0-s003.docx]

**Appendix S3**
Quality appraisal using Mixed Method Appraisal Tool (MMAT)

After two screening questions, this tool allows the appraisal of five categories of study designs: qualitative, quantitative randomized controlled trials, quantitative non-randomized, quantitative descriptive and mixed methods. Each category had five criteria questions in which “yes”, “no” or “can’t tell” as response was possible, resulting in a possible score of 0-100% of the quality criteria met.

| **Study** | **Methodological quality criteria** | | | | | **Comments** |
| --- | --- | --- | --- | --- | --- | --- |
| **2. Quantitative randomized controlled trials** | **2.1** | **2.2** | **2.3** | **2.4** | **2.5** |  |
| Abdel-Ghaffar, 2019 | Yes | Yes | Yes | Yes | Yes |  |
| Bernardis, 2016 | Yes | Can’t tell | Yes | No | Yes | Limited baseline data reported. Outcome assessors not blinded. |
| Butwick, 2007 | Yes | Yes | Yes | Can’t tell | Yes | Limited information about blinding. |
| deFigueiredoLocks, 2012 | Can’t tell | Can’t tell | Yes | Yes | Yes | Limited information about randomization and baseline data. |
| Haim, 2024 | Yes | Yes | Yes | Yes | Yes |  |
| Hong, 2005 | Yes | Can’t tell | Yes | Yes | Yes | Limited baseline data reported. |
| Hui, 2006 | Yes | Yes | Yes | Yes | Yes |  |
| Kholeif , 2024 | Yes | Yes | Yes | No | Yes | Outcome assessors not blinded. |
| Munday…Gosden, 2018 | Yes | Yes | Yes | Yes | No | 13 protocol deviations. |
| Sadiq, 2025 | Yes | Can’t tell | Yes | No | Yes | Limited baseline data reported. Outcome assessors not blinded |
| Sween, 2021 | Yes | Can’t tell | Yes | Yes | Yes | Limited baseline data reported. |
| TanSookKuan, 2023 | Yes | Yes | Yes | Yes | Yes |  |
| Techanivate, 2005 | Yes | Can’t tell | Yes | Yes | Yes | Limited baseline data reported. |
| **3. Quantitative non-randomized** | **3.1** | **3.2** | **3.3** | **3.4** | **3.5** |  |
| Hoefnagel, 2020 | No | Yes | Yes | Can’t tell | Yes | Only repeat caesarean section patients and the rationale not clarified. |
| Ismaiel, 2025 | Yes | Yes | Yes | Can’t tell | Yes | No description of possible confounders. |
| Kavee, 1991 | Can’t tell | Yes | Can’t tell | Can’t tell | Can’t tell | Very limited description of the methodology of the study. |
| Marin, 2022 | Yes | Yes | Yes | Can’t tell | Yes | No description of possible confounders. |
| Munday…Yates, 2018 | Can’t tell | Yes | Yes | No | Yes | No report of exclusion criteria. Substantial missing data of active warming. |
| Riley, 2019 | Yes | Yes | Yes | Can’t tell | Yes | No description of possible confounders. |
| **4. Quantitative descriptive** | **4.1** | **4.2** | **4.3** | **4.4** | **4.5** |  |
| Burey, 2021 | Yes | Can’t tell | Yes | Can’t tell | Can’t tell | Very limited description of the methodology of the study. |
| Hess, 2005 | Yes | Can’t tell | Yes | Yes | Yes | No report of inclusion or exclusion criteria. |
| Hilton, 2016 | Can’t tell | Yes | Yes | Yes | Yes | Very long inclusion period, however a small number of patients included. |
| Munday, 2023 | Yes | Can’t tell | Can’t tell | No | Can’t tell | Limited methodology section. Only included patients with reported symptoms. |
| Murphey, 2022 | Yes | Yes | Yes | Yes | Yes |  |
